# Curious Veterinary Experience

**Published:** 1881-07

**Authors:** John N. Navin

**Affiliations:** Veterinary Editor of Indiana Farmer; Indianapolis, Ind.


### A CURIOUS VETERINARY EXPERIENCE—BLOOD-LETTING IN THE WEST.

EDITOR OF JOURNAL OF COMPARATIVE MEDICINE:—

I was recently called by telegram to visit a sick horse near Rockville, Park County, this State. On arriving at the place, and on my way to the owner's residence, I inquired of the man who took me in a buggy from the train, something about the horse's complaint.

"Well," said he, "the V. S. calls it the 'hooks,' and says that the horse turns his eyes unnaturally upward."

I remarked to the young man that I had no need to go farther, for that the doctor—as is usual out here—must have cut the haw or haws out of his eyes. "No," said he, "but he bled him."

"Scarified his eyelids, I suppose?"

"Not at all, sir; he bled him nine gallons from his neck."

Never supposing that any man would be guilty of such barbarous ignorance in this, the nineteenth century, I remarked that nine quarts was pretty heavy bleeding, especially for an inflammation of the haw.

Supposing, I presume, that I must be a novice in the art of bleeding, as practised in Park County, he remarked with emphasis: "Not nine quarts, but *thirty-six quarts*, and he returned next day and drew nine quarts more. That happened day before yesterday; and he came again this morning to bleed him again; but my brother, who is owner of the horse, told him that he had become skeptical about bleeding, and had telegraphed for Dr. Navin, of Indianapolis, who would arrive on the next train, and wished him to wait and consult over the case."

When I arrived I found the horse to be suffering from a slight attack of tetanus, and very weak from loss of blood. He was so nervous that I administered opiates through the rectum, which, with lobelia, was the treatment I ordered.

On the fourth day after this, I received a letter announcing that his jaws were relaxed, and his eyes much better. Some days later I received another letter, stating that he showed no further symptoms of the disease, but was still weak, and that his jaws were swelling. I ordered diffusible stimulants, diuretics, and a little sulphur, with warmth to the skin, together with the application of the tincture of iodine to the jaws, twice daily.

If you are as ignorant of the disease called out here the "hooks," as I was when I first came to this country, I must inform you that an inflammation of the haw, or any ailment that causes the horse to withdraw the eye from strong rays of light, constitutes a case of "hooks." The proprietor of a horse so affected sees that something is wrong, and very properly seeks the assistance of the best veterinary skill within reach. The usual result is as follows:

A veterinary surgeon is summoned, and joins a crowd of the neighbors, whose skill has been previously exhausted. Conscious of the superiority of his skill, he struts about a little and pronounces it a dangerous case of "hooks," and for the purpose of securing a five or ten dollar fee, proposes to bet a V that the haw is turning into bone, and that unless it is excised it will penetrate the eye, etc. The job secured, he casts the horse, and as these fellows are generally armed with a curved needle and thread, he runs the thread through the haw pretty deeply, and cuts both the membrane and the cartilage clean out, and with the impudence of Lucifer with all his host, presents the so-called bone, and reminds his patron of his pathological prophecy and skill. "Just as I told you, there is the bone!" Thus, he not only excites the admiration of the proprietor of the much-injured dumb animal, but also that of the entire crowd, and securing their future employment of his services.

The above is a specimen of Western Veterinary Surgery.

JOHN N. NAVIN, D. V. S.,
*Veterinary Editor of Indiana Farmer.*

INDIANAPOLIS, Ind., June 10, 1881.

